# A case report of disseminated histoplasmosis not responding to primary treatment by itraconazole

**DOI:** 10.22034/cmm.2025.345248.1613

**Published:** 2025-05-22

**Authors:** Prashant Gupta, Kalpana Kuntal, Nishant Gupta, Anand Srivastava

**Affiliations:** 1 Department of Microbiology, King George’s Medical University, Lucknow, India; 2 Department of Pathology, King George’s Medical University, Lucknow, India; 3 Department of Respiratory Medicine, King George’s Medical University, Lucknow, India

**Keywords:** Dimorphic fungus, Disseminated histoplasmosis, Itraconazole, Liposomal amphotericin B, Urinary *Histoplasma* antigen

## Abstract

**Background and Purpose:**

Histoplasmosis, caused by *Histoplasma capsulatum*, typically presents as a pulmonary infection but can disseminate, with oral lesions being common among immunocompromised individuals. However, this is rare among immunocompetent patients. Preferred treatments include itraconazole for mild cases and liposomal amphotericin B for severe forms.

**Case presentation:**

This study aimed to report a 28-year-old female who developed disseminated histoplasmosis following a right oroantral fistula after dental surgery. It was initially misdiagnosed as Actinomycosis; however, a positive urinary *Histoplasma* antigen test confirmed histoplasmosis. Despite itraconazole therapy (200 mg twice daily, later increased to 600 mg), her condition continued to deteriorate, with disease progression seen on imaging. Switching to six weeks of intravenous liposomal amphotericin B led to marked improvement, resolution of lung nodules, and negative antigen testing. She was discharged with a 12-month course of itraconazole therapy.

**Conclusion:**

This case highlights the importance of timely recognition and adjustment of treatment in non-severe histoplasmosis, particularly for patients who do not respond adequately to itraconazole therapy.

## Introduction

Histoplasmosis is a systemic fungal infection caused by the dimorphic fungus *Histoplasma capsulatum*, which commonly presents as a self-limiting pulmonary infection or in disseminated form. A recognized manifestation of disseminated histoplasmosis is the presence of oral lesions, observed in 25-45% of immunocompromised individuals [ 1 ]. These lesions can range from nodules to painful shallow or deep ulcers. Presentation of disseminated histoplasmosis with oral lesions is rare among immunocompetent patients [ 2 ]. Although acute pulmonary histoplasmosis typically resolves on its own, itraconazole is advised for mild to moderate cases, while severe cases may require lipid formulations of amphotericin B [ 3 ]. However, issues related to oral bioavailability, potential drug interactions, and tolerability limit the contemporary use of these treatments. While therapeutic drug monitoring (TDM) of itraconazole is highly recommended, it is not always accessible [ 4 ]. The present study aimed to report a case whose disease progressed under itraconazole therapy but responded to liposomal amphotericin B (LAMB). 

## Case Presentation

This study reports a case of disseminated histoplasmosis in a 28-year-old female, who developed discharge from her right upper molar with right facial pain and swelling, and right-sided nasal bleeding two weeks after dental surgery (right upper molar tooth extraction). The patient was diagnosed with a right-sided oro-antral fistula, which was repaired via the Caldwell luc approach under general anesthesia. All initial routine blood test results were normal. Abdominal and pelvis ultrasonography detected no significant abnormality. Biopsy findings were suggestive of granulomatous inflammation, and the patient was diagnosed with Koch’s sinus. The patient underwent 13 months of anti-tubercular therapy, without clinical improvement. Simultaneously, the patient experienced watering eye and pain on the right side of the face. Non-contrast Computed Tomography of paranasal sinuses revealed a bony defect in the floor of the right orbit, along with a solitary nodule (22×20 mm) and lung imaging showed internal necrosis and tiny eccentric cavitation in the posterobasal segment of the right lower lobe. A biopsy from the right maxillary sinus suggested actinomycosis, leading to the initiation of intravenous ceftriaxone at 2 g twice daily for 45 days; however, no improvement was observed. Due to the lack of response to ceftriaxone, multiple reviews were conducted. A biopsy of the lung nodule was inconclusive, but the urinary *Histoplasma* antigen test came positive. The patient denied any recent travel history or any exposure to bird or bat droppings, caves, or significant amounts of soil that might have exposed the patient to microconidia. Given the rarity of disseminated *histoplasmosis* in immunocompetent individuals, a comprehensive immunodeficiency evaluation, including tests for human immunodeficiency virus (HIV) 1/2 antibodies and HBsAg, was performed, with negative results. 

**Figure 1:**
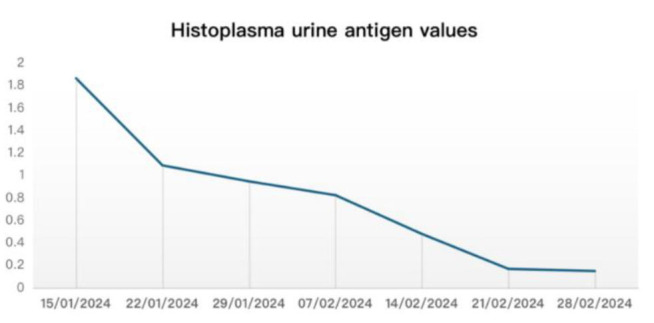
Weekly *Histoplasma* urine antigen values

**Figure 2:**
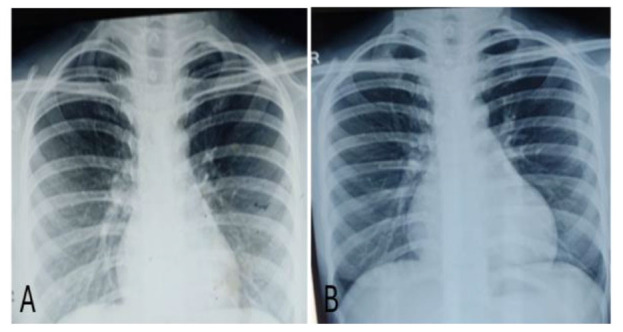
X-ray posteroanterior view before (A) and after (B) starting liposomal amphotericin B therapy

**Figure 3:**
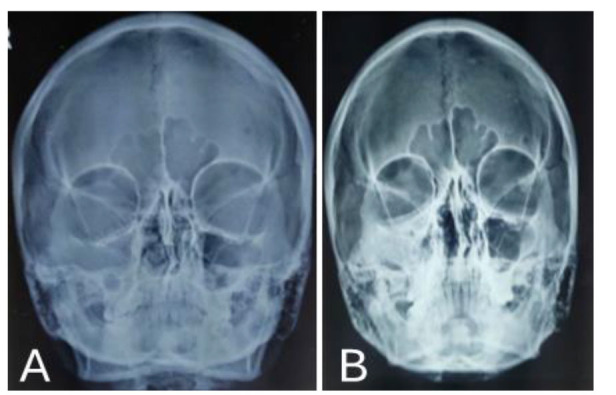
X-ray of paranasal sinuses before (A) and after (B) starting liposomal amphotericin B therapy

Therefore, the patient was started on itraconazole 200 mg twice daily for 4 months. On reviewing the right maxillary histopathological examination, the patient was diagnosed with histoplasmosis, and the CT scan showed an increased dimension of orbital soft tissue and the presence of additional lung nodules. Functional endoscopic sinus surgery was performed on the right side under general anesthesia and was repeated after 45 days, owing to an increase in the dimension of the orbital soft tissue on a follow-up CT scan. However, TDM on three different occasions showed drug levels below the therapeutic range (Table 1). Therefore, the dose was increased to 600 mg per day, administered in three divided doses. Repeat TDM levels for itraconazole were within the therapeutic range. However, follow-up contrast-enhanced computed tomography of the paranasal sinuses and chest revealed an increase in the size of the orbital tissue and the initial lung nodule (27×22 mm), along with the appearance of a subcentric new nodule in the adjacent lung segment. *Histoplasma* urine antigen values also came high (4.03 ng/ml). Itraconazole was stopped and the patient was started on LAMB 200 mg I/V, for 6 weeks which was stopped only after two consecutive negative *Histoplasma* urine antigen values. Kidney function tests, serum electrolytes, and serum magnesium levels were monitored on alternate days, with all values remaining within the normal range. *Histoplasma* urine antigen values were repeated on a weekly basis (Figure 1). Serial monitoring of chest X-ray posteroanterior view and X-ray paranasal sinuses were performed on a weekly basis (Figure 2 and 3). Radiological findings improved and nodules disappeared. Simultaneous urinary *Histoplasma* antigen also came negative. The patient was discharged and achieved full recovery after taking itraconazole 200 mg orally twice daily for 12 months.

**Table 1:** Plasma itraconazole levels during the course of illness

Plasma itraconazole levels during the initial 6 months of therapy		
Date	Itraconazole dosage	Plasma itraconazole concentration, µg/ml (therapeutic range, µg/ml)
August, 2023	200 mg twice daily	0.784
September, 2023	200 mg twice daily	0.406
October, 2023	200 mg twice daily	Nil
**Plasma Itraconazole levels during 12 months after discharge**		
February, 2024	200 mg twice daily	0.074
April, 2024	200 mg twice daily	1.795

## Discussion

*Histoplasma capsulatum* is found in soils worldwide, particularly in the Midwest of the United States, as well as in Central and South America [ 5 ]. In India, a review of reports from 1994 to 2017, which included 144 cases of histoplasmosis recorded after 1954, showed a widespread distribution. Most cases were clustered in small areas of West Bengal and Assam, the Gangetic Plains, and Western India, with sporadic occurrences in the southern and a few northern states [ 6 , 7 ]. This fungus thrives in nitrogen-rich soils, often those enhanced by bird or bat droppings. It exhibits thermal dimorphism, existing as a filamentous mold at temperatures below 35 °C and as a yeast at temperatures above this threshold. Humans can become infected by inhaling microconidia and mycelial fragments, with an incubation period of 1-3 weeks [ 8 ]. Most individuals exposed to low levels of *H. capsulatum* remain asymptomatic; however, acute pulmonary histoplasmosis can occur when matter containing *H. capsulatum* is disrupted, thus creating an infective aerosol that enters the respiratory system [ 90 ].

Histoplasmosis can present in three primary forms: pulmonary, progressive disseminated, and primary cutaneous forms [ 10 ]. Severity of the disease depends on the immune status of the host. Immunocompromised individuals, children (<2 years of age), and the elderly are more susceptible to developing symptomatic disease. Progressive disseminated histoplasmosis is marked by the persistent growth of *H. capsulatum* in various organs and can arise from the reactivation of latent infections or from exposure to a high concentration of fungal spores. While this severe form often affects immunocompromised individuals or those at the extremes of age, even immunocompetent patients can experience infections, typically presenting with nonspecific symptoms, such as prolonged fever, weight loss, oropharyngeal ulcers, hepatosplenomegaly, and lymphadenopathy. However, it is often misdiagnosed and treated as tuberculosis prior to the correct diagnosis, as occurred in our case [ 11 ]. 

Diagnosis of histoplasmosis can be particularly difficult in immunocompetent patients. However, a combination of strong clinical suspicion, relevant symptoms, imaging studies, and urine tests for *Histoplasma* antigen can be helpful. The immunocompetent status, histopathology findings of tissue biopsy, and the rare occurrence of *H. capsulatum* in India complicated the diagnosis in this patient. After the urinary *Histoplasma* antigen test came positive, the patient was suspected of Histoplasmosis and started on itraconazole. However, after six weeks of therapy, the clinical response of the patient was deemed suboptimal. This was characterized by the increased dimension of orbital soft tissue and the emergence of an additional lung nodule, indicating the progression of the disease despite treatment.

Therapeutic drug monitoring showed that plasma levels of itraconazole were below the therapeutic range. Despite an increased dosage to 600 mg, the itraconazole levels, while consistently above the therapeutic thresholds, did not lead to the expected clinical response. As a result, the disease was regarded as refractory to itraconazole. Ultimately, the patient was switched to the LAMB 200 mg intravenously once daily. Itraconazole has established itself as a cornerstone in the treatment of mild to moderate histoplasmosis, offering a generally favorable safety profile and effective outcomes. However, a review of the literature identifies patients with disseminated histoplasmosis who have experienced relapses while undergoing itraconazole therapy [ 12 , 13 ]. Potential contributing factors include patient non-compliance and drug intolerance, which can complicate the treatment course and hinder therapeutic success. 

Given the diverse clinical presentations of histoplasmosis and the varying patient responses to treatment, the selection of an appropriate therapy should be tailored to the individual. In this case, the switch to LAMB at a dose of 200 mg intravenously once daily proved to be a pivotal decision. Following a rigorous 6-week course, the patient demonstrated significant clinical improvement, and radiological evaluations confirmed the resolution of nodules that were previously evident. Results of this case are consistent with those of a study performed by Wheat et al., who compared the efficacy of LAMB and itraconazole in the clearance of fungal burden in HIV patients with disseminated histoplasmosis. The findings indicated that among patients with positive blood cultures at baseline and accessible follow-up cultures, those treated with LAMB experienced a more rapid clearance of fungemia, compared to those treated with itraconazole. Additionally, while antigen concentrations also declined more quickly with LAMB, the reduction was less pronounced than the clearance of fungemia [ 14 ]. 

## Conclusion

This case of disseminated histoplasmosis in an immunocompetent individual not only emphasizes the need for a strong clinical suspicion of histoplasmosis as a differential diagnosis to avoid misdiagnosis but also demonstrates the challenges posed by itraconazole therapy in terms of drug monitoring and efficacy. The favorable response to LAMB after itraconazole failure highlights the importance of individualized treatment plans, especially in refractory cases. Addressing the barriers to timely diagnosis and continued research is necessary to improve diagnostic methods, therapeutic drug monitoring access, and alternative treatment strategies for histoplasmosis, ensuring better outcomes for patients.
